# Dietary Inflammatory Index and female infertility: findings from NHANES survey

**DOI:** 10.3389/fnut.2024.1391983

**Published:** 2024-09-19

**Authors:** Wenhui Wang, Yuxiao Dong, Kun Wang, Heming Sun, Huan Yu, Bin Ling

**Affiliations:** ^1^Department of Gynecologic Oncology, Beijing Obstetrics and Gynecology Hospital, Capital Medical University, Beijing Maternal and Child Health Care Hospital, Beijing, China; ^2^Department of Obstetrics and Gynecology, China-Japan Friendship Hospital, Beijing, China; ^3^Beijing Chengshou Temple Community Health Service Center, Beijing, China; ^4^Institute of Clinical Medical Sciences, China-Japan Friendship Hospital, Beijing, China

**Keywords:** infertility, Dietary Inflammatory Index, diet, inflammation, NHANES

## Abstract

**Background and objectives:**

Infertility is a pressing public health concern on a national scale and has been linked to inflammatory conditions. However, limited research has been conducted on the impact of the Dietary Inflammatory Index (DII) on female infertility. This study sought to investigate the association between DII and infertility utilizing data from the National Health and Nutrition Examination Survey (NHANES).

**Methods:**

This cross-sectional study included a cohort of 3,071 women aged 20–44 years from three NHANES cycles (2013–2018). Dietary information was collected to calculate the Dietary Inflammatory Index (DII), while infertility status was determined through positive responses to specific questions in a questionnaire. The association between DII scores and infertility was assessed using adjusted multivariate logistic regression analyses. Subgroup analysis and restricted cubic spline (RCS) was conducted for further investigation.

**Results:**

Among the participants, 354 women (11.53%) were identified as experiencing infertility. Upon adjusting for all covariates, a positive correlation was observed (OR = 1.61, 95% CI: 1.12–2.31). Individuals with DII scores in the highest quartile exhibited significantly greater odds of infertility compared to those in the lowest quartile (OR = 1.71, 95% CI = 1.17–2.51). The relationship between DII and infertility in the RCS models demonstrated an S-shaped curve. When using the median DII as a reference point, a higher DII was associated with an increased prevalence of infertility. Additionally, obesity was found to be a significant factor.

**Conclusions:**

Our research indicated that the DII was positively correlated with an increased likelihood of infertility in American women among the ages of 20 and 44. These results contribute to the existing literature and underscore the need for further validation through larger prospective cohort studies.

## Introduction

Infertility is a medical condition characterized by the inability to achieve a clinical pregnancy following 1 year of unprotected sexual intercourse (1). It impacts ~15% of couples of reproductive age worldwide, resulting in significant economic and psychological consequences for society (2). The U.S. Centers for Disease Control (CDC) have identified the diagnosis and treatment of infertility as a priority in public health (3). Research suggests that inflammatory conditions like polycystic ovary syndrome (PCOS) and endometriosis may be connected to infertility, and anti-inflammatory treatments have been shown to improve pregnancy outcomes in affected women (4–6).

Evidence suggests that adopting healthy dietary patterns such as the Healthy Nordic Diet, the Okinawan diet, and the Mediterranean Diet, which are known to reduce inflammation, can have positive effects on fertility outcomes. Conversely, Western dietary patterns characterized by high intake of saturated fat, refined carbohydrates, and animal proteins are associated with increased inflammation and adverse pregnancy outcomes (7). Various nutrients like vitamins, minerals, fatty acids, phytochemicals, and non-nutritive compounds like carotenoids and flavonoids can modulate inflammatory processes. Recent research indicates that diet can impact inflammation and infertility by influencing the gene function, gut microbiome composition, BMI, and other factors (7).

The impact of diet on inflammatory potential can be assessed using the Dietary Inflammatory Index (DII), a validated dietary index derived from literature to measure inflammatory potential (8). The DII has been shown to correlate with systemic inflammation levels and is closely linked to the expression of various blood inflammatory markers such as C-reactive protein (CRP), tumor necrosis factor (TNF)-a, interleukin (IL)-1β, IL-6, and IL-10 (9). It is widely utilized to investigate the relationship between diet-induced inflammation and the development of conditions like metabolic syndrome, cardiovascular disease, and cancer (10).

However, the relationship between the DII and infertility remains inadequately understood, and there is limited knowledge regarding its potential as an assessment tool for infertility. We hypothesized that DII serves as a predictor for infertility risk. This study aims to explore the association between DII and infertility, offering insights for the treatment and management of infertility. We conducted a national population-based survey utilizing data from three cycles of the National Health and Nutrition Examination Survey (NHANES) conducted between 2013 and 2018, which included representative samples of American civilian women.

## Methods

### Data source and study population

A cross-sectional study was conducted using data from the NHANES cycles between 2013 and 2018 to evaluate the health and nutritional status of adults and children in the United States. NHANES employs complex stratified sampling methods in a multistage study to gather a representative sample of the U.S. population every 2 years. Approval for all protocols was obtained from the National Center for Health Statistics institutional review board, and participants were required to provide informed consent.

**Figure 1:**
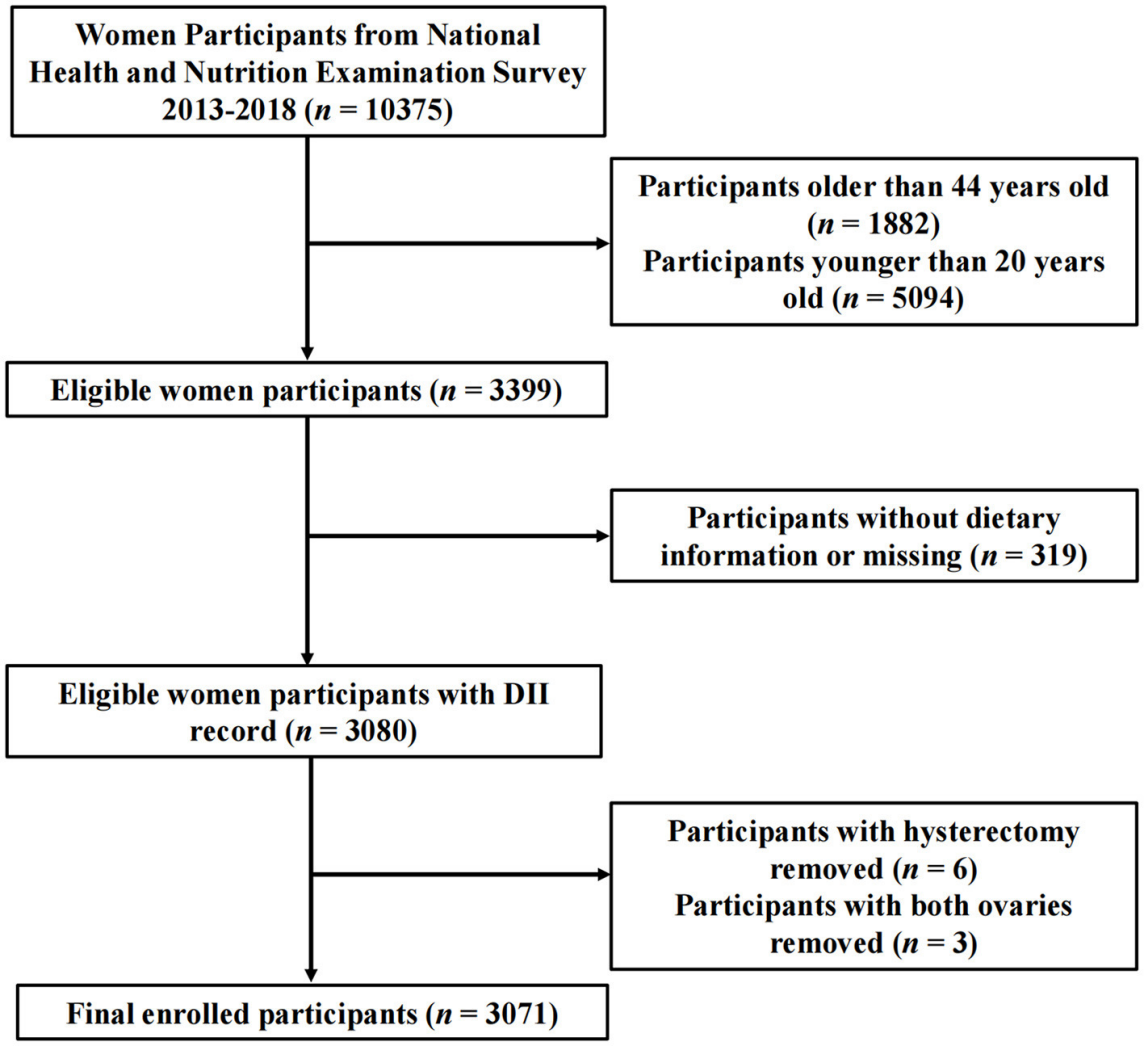
Participants flow chart.

This study specifically targeted female respondents aged 20–44 years, as this age range already has available data on reproductive health. Women who did not provide dietary information were excluded from the study. Additionally, those who had undergone hysterectomy or bilateral oophorectomy were also excluded, as they may not have had the experience of trying to conceive. A total of 3,071 participants were included in the complete case analysis. The process of sample selection is detailed in Figure 1.

### The definition of DII

Dietary Inflammatory Index (DII) serves as a valuable tool for assessing the inflammatory potential of an individual's diet (10). By conducting a 24-hour dietary recall interview at a mobile examination center, data on 27 components of each participant's daily food intake were collected. The DII score, reflecting the inflammatory properties of the diet, was calculated using a built-in function in the “nhanesR” package. The Nutrition Methodology Working Group of the NHANES conducted comprehensive dietary recall interviews to gather detailed dietary information. Initially, face-to-face interviews were conducted in the Mobile Examination Center (MEC), followed by a second review via telephone. For this study, we opted to utilize the average of two dietary interviews as a representation of an individual's daily dietary intake for calculating the Dietary Inflammatory Index (DII). All food components available in the NHANES database were considered in the DII calculation, with each component being assigned a specific DII score based on its impact on six major inflammatory biomarkers: IL-1β, IL-4, IL-6, IL-10, TNF-α, and CRP. Utilizing this scoring system, the DII for each food component was computed. To evaluate the overall impact of all dietary intakes on inflammation for a participant within a day, individual DII scores for all food components were summed to derive the participant's overall DII. This involved standardizing each food parameter, converting it to a *Z*-score, and adjusting for centered proportions based on the inflammatory effect index. The resulting DII score provided insight into the inflammatory nature of each participant's diet (11). Higher DII scores signify a more pro-inflammatory diet, while lower scores indicate a more anti-inflammatory diet. In our analysis, we treated the DII score as a continuous variable and divided the total sample into quartiles (Q1, Q2, Q3, and Q4) for further examination.

### The definition of infertility

According to the definition of infertility (24), women who answered “yes” to either of the following questions were considered ever infertile: “Have you ever attempted to become pregnant over a period of at least a year without becoming pregnant?” or “Have you ever been to a doctor or other medical provider because you have been unable to become pregnant?”

### Covariates

The selection of covariates in this study was based on professional judgment and informed by previous research (12, 24). The included covariates encompassed factors such as age, race/ethnicity, education, BMI, marital status, family income, menstrual periods, pelvic infection, as well as history of female hormone use, birth control pill use, smoking, and alcohol consumption. These covariates were sourced from the demographic, examination, reproductive health questionnaire, and smoking questionnaire sections of the NHANES database.

### Statistical analysis

All statistical analyses were performed following the NHANES analytic guidelines. A descriptive analysis was carried out on the demographic and measurement indicators of the study population, categorized into two groups based on infertility status. Continuous variables were presented as mean ± standard deviation for normal distributions, median with interquartile range for skewed distributions, or percentages for categorical variables. χ^2^-tests were used for categorical variables, two independent sample *t*-tests for normally distributed variables, and Wilcoxon rank sum test for variables not following a normal distribution to assess differences among the groups.

The association between DII and infertility was evaluated through two logistic regression models: a crude model with no covariate adjustment and an adjusted model including all covariates. To account for the impact of age on infertility, a subgroup analysis stratified by age (35 years) was conducted. Odds ratios (OR) with 95% confidence intervals (CI) were calculated to measure the strength of the association. Additionally, a restricted cubic spline (RCS) regression model was used to further explore the relationship between DII and infertility. This model included four knots at the 5th, 35th, 65th, and 95th percentiles of DII, with the median DII serving as the reference point. Single-factor logistic regression analysis was initially performed in a crude model, followed by a multi-factor logistic regression analysis in model 1 and model 2. Two models were used in the analysis: Model I adjusted for age, marital status, and race/ethnicity, while Model II further adjusted for education levels, family income, BMI, regular menstrual periods, pelvic infection, female hormones taken, birth control pills taken, drinking history, and smoking history. Additionally, the subgroup analysis was conducted based on age, race/ethnicity, and BMI. Statistical analyses were carried out using SAS software version 9.4, with a significant level set at *p* < 0.05.

## Results

### Population characteristics of participants

A total of 3,071 women were included in this study, consisting of 354 infertile women and 2,717 control women. The basic characteristics of the study population are presented in Table 1. Infertile women exhibited significantly higher age and BMI compared to control women (*P* < 0.05). Detailed baseline clinical characteristics categorized by DII score quartiles are provided in Supplementary Table 1.

**Table 1:** Baseline characteristics.

	**Overall (*n* = 3,071)**	**DII-Q1**	**DII-Q2**	**DII-Q3**	**DII-Q4**	***P*-value**
Age, years	31.18 [30.80, 31.57]	31.65 [31.04, 32.26]	30.80 [29.92, 31.68]	31.33 [30.69, 31.98]	30.91 [30.45, 31.38]	0.21
**Race/ethnicity**						< 0.001^***^
White	56.38 [48.75, 64.00]	57.93 [52.95, 62.91]	55.87 [50.00, 61.75]	57.27 [50.16, 64.37]	54.16 [48.41, 59.92]	
Black	13.26 [10.81, 15.71]	9.27 [6.58, 11.95]	11.40 [8.51, 14.30]	14.10 [10.98, 17.23]	18.86 [14.53, 23.20]	
Mexican	11.79 [8.88, 14.71]	13.06 [9.56, 16.57]	14.93 [10.74, 19.12]	9.86 [6.79, 12.93]	9.09 [6.27, 11.90]	
Other Hispanic	8.05 [6.33, 9.77]	8.44 [6.67, 10.20]	7.39 [5.30, 9.49]	8.01 [5.00, 11.02]	8.39 [5.81, 10.96]	
Others	10.52 [8.98, 12.06]	11.30 [8.51, 14.09]	10.40 [8.49, 12.32]	10.76 [7.38, 14.13]	9.50 [7.39, 11.61]	
**Education levels**						< 0.001^***^
Less than high school	3.26 [2.37, 4.16]	3.04 [1.74, 4.35]	3.22 [1.99, 4.45]	4.14 [2.62, 5.67]	2.56 [1.38, 3.73]	
High school or equivalent	28.03 [24.70, 31.36]	18.35 [14.81, 21.89]	27.62 [23.86, 31.38]	27.14 [22.33, 31.95]	40.47 [34.76, 46.18]	
College or above	68.68 [61.84, 75.52]	78.61 [74.37, 82.84]	69.16 [64.82, 73.50]	68.72 [63.40, 74.03]	56.97 [51.27, 62.68]	
**Marital status**, ***n*** **(%)**						0.02^*^
Divorced	6.09 [4.91, 7.27]	5.85 [3.49, 8.21]	5.48 [3.54, 7.43]	5.68 [3.37, 7.99]	7.49 [5.47, 9.51]	
Living with partner	14.69 [12.68, 16.69]	12.14 [8.67, 15.61]	14.72 [11.90, 17.53]	15.13 [11.55, 18.72]	17.02 [13.91, 20.12]	
Married	44.16 [39.56, 48.76]	50.96 [46.35, 55.57]	44.15 [38.84, 49.46]	43.65 [38.89, 48.41]	37.08 [32.31, 41.85]	
Never married	31.62 [28.52, 34.72]	28.69 [24.52, 32.87]	33.18 [28.57, 37.79]	31.32 [27.45, 35.19]	33.54 [29.04, 38.04]	
Separated	3.18 [2.48, 3.87]	2.11 [1.05, 3.18]	2.12 [1.25, 2.99]	3.93 [2.44, 5.42]	4.69 [3.03, 6.35]	
Widowed	0.27 [0.07, 0.47]	0.24 [0.04, 0.52]	0.36 [0.04, 0.67]	0.28 [0.11, 0.68]	0.19 [0.08, 0.46]	
**Family income**						0.002^**^
< 2,000$	17.44 [15.42, 19.46]	13.66 [10.84, 16.49]	16.79 [13.53, 20.04]	20.14 [16.91, 23.38]	22.32 [18.21, 26.42]	
≥2,000$	78.76 [72.28, 85.23]	86.34 [83.51, 89.16]	83.21 [79.96, 86.47]	79.86 [76.62, 83.09]	77.68 [73.58, 81.79]	
BMI, kg/m^2^	29.38 [28.92, 29.84]	28.02 [27.30, 28.74]	29.21 [28.28, 30.13]	29.70 [29.03, 30.38]	30.73 [29.92, 31.54]	< 0.001^***^
Regular menstrual periods, (%)	90.12 [83.59, 96.64]	93.21 [90.47, 95.94]	90.58 [87.23, 93.92]	90.89 [88.16, 93.62]	85.24 [82.86, 87.62]	0.01^*^
Pelvic infection, (%)	4.47 [3.46, 5.48]	2.74 [1.28, 4.21]	4.65 [3.10, 6.21]	4.67 [2.79, 6.54]	6.10 [4.24, 7.97]	0.06
Female hormones taken, %	4.46 [3.11, 5.81]	3.96 [1.91, 6.01]	4.65 [2.29, 7.02]	5.08 [2.48, 7.68]	4.16 [2.08, 6.23]	0.86
Birth control pills taken, %	72.92 [66.43, 79.41]	75.76 [72.21, 79.31]	72.90 [69.15, 76.65]	73.84 [70.47, 77.21]	69.01 [64.80, 73.21]	0.05^*^
Smoking, %	19.75 [17.29, 22.22]	12.00 [9.27, 14.72]	14.36 [11.16, 17.56]	23.10 [18.63, 27.57]	30.69 [26.61, 34.77]	< 0.001^***^
Drinking, %	83.64 [77.24, 90.04]	87.35 [83.52, 91.18]	86.77 [83.49, 90.05]	86.38 [82.34, 90.41]	85.44 [82.41, 88.47]	0.84

Continuous variables are presented as the mean and 95% confidence interval, category variables are presented as the proportion and 95% confidence interval.

BMI, body mass index.

^***^P-value < 0.001.

^**^P-value < 0.01.

^*^P-value < 0.05.

### Association of DII with infertility

Table 2 illustrates the comparison of DII components between the non-infertility group and the infertility group. The Wilcoxon rank sum test revealed that the average DII in infertile women was significantly higher than in the control group [2.01 (1.80, 2.21) vs. 1.73 (1.59, 1.87), *P* = 0.03]. Multivariate regression analysis indicated a positive correlation between DII score and infertility (OR = 1.09; 95% CI = 1.01–1.18; Table 3). Q4 showed a significantly higher risk of infertility compared to Q1 in various models, with a 71% increased likelihood of infertility observed when comparing Q4 to Q1 (OR = 1.71; 95% CI = 1.17–2.51).

**Table 2:** Comparison of DII components between non-infertility group and infertility group.

**Variables**	**Overall (*n* = 3,071)**	**Non-infertility (*n* = 2,717)**	**Infertility (*n* = 354)**	***P*-value**
DII	−0.03 [−0.04, −0.03]	1.73 [1.59, 1.87]	2.01 [1.80, 2.21]	0.03^*^
Vitamin A	0.35 [0.33, 0.37]	0.20 [0.19, 0.21]	0.22 [0.20, 0.24]	0.05^*^
Vitamin B1	0.00 [0.00, 0.00]	0.03 [0.03, 0.03]	0.03 [0.02, 0.04]	0.53
Vitamin B2	0.05 [0.05, 0.06]	0.00 [−0.01, 0.00]	0.00 [−0.01, 0.00]	0.43
Vitamin B6	0.13 [0.12, 0.13]	−0.06 [−0.07, −0.04]	−0.03 [−0.06, −0.01]	0.14
Vitamin B12	0.23 [0.21, 0.24]	−0.03 [−0.04, −0.03]	−0.03 [−0.04, −0.03]	0.93
Vitamin C	0.25 [0.24, 0.27]	0.22 [0.20, 0.24]	0.26 [0.23, 0.29]	0.04^*^
Vitamin D	0.09 [0.08, 0.11]	0.25 [0.24, 0.27]	0.26 [0.23, 0.29]	0.51
Vitamin E	0.10 [0.09, 0.12]	0.09 [0.07, 0.11]	0.11 [0.07, 0.16]	0.37
Iron	0.05 [0.03, 0.06]	−0.01 [−0.01, −0.01]	−0.01 [−0.01, 0.00]	0.85
Magnesium	−0.01 [−0.01, −0.01]	0.10 [0.08, 0.11]	0.13 [0.10, 0.16]	0.1
Zinc	−0.09 [−0.09, −0.08]	0.04 [0.03, 0.06]	0.06 [0.02, 0.09]	0.46
Selenium	0.08 [0.08, 0.08]	−0.08 [−0.09, −0.08]	−0.09 [−0.10, −0.07]	0.88
Total fatty acid	−0.09 [−0.10, −0.08]	0.01 [0.00, 0.02]	0.02 [0.00, 0.05]	0.28
Total saturated fatty acid	0.00 [0.00, 0.00]	−0.10 [−0.11, −0.09]	−0.07 [−0.10, −0.03]	0.08
MUFA	−0.07 [−0.08, −0.06]	0.00 [0.00, 0.00]	0.00 [0.00, 0.00]	0.14
PUFA	−0.03 [−0.03, −0.02]	−0.07 [−0.08, −0.06]	−0.08 [−0.11, −0.05]	0.6
n3 Polyunsaturated fatty acid	0.28 [0.27, 0.28]	0.28 [0.27, 0.28]	0.27 [0.27, 0.28]	0.4
n6 Polyunsaturated fatty acid	−0.06 [−0.07, −0.06]	−0.06 [−0.07, −0.06]	−0.06 [−0.07, −0.05]	0.58
Cholesterol	0.21 [0.19, 0.22]	−0.03 [−0.03, −0.03]	−0.02 [−0.03, −0.01]	0.29
Folate	−0.03 [−0.04, −0.03]	0.13 [0.12, 0.13]	0.13 [0.11, 0.14]	0.92
β-Carotene	0.03 [0.03, 0.03]	0.35 [0.32, 0.37]	0.38 [0.33, 0.43]	0.18
Niacin	−0.05 [−0.07, −0.04]	0.05 [0.05, 0.06]	0.05 [0.03, 0.06]	0.76
Energy	0.00 [−0.01, 0.00]	−0.03 [−0.04, −0.03]	−0.02 [−0.04, −0.01]	0.37
Protein	−0.04 [−0.04, −0.03]	0.00 [−0.01, 0.00]	0.00 [−0.01, 0.00]	0.9
Carbohydrate	0.24 [0.21, 0.27]	−0.04 [−0.04, −0.03]	−0.04 [−0.04, −0.03]	0.94
Dietary fiber	0.01 [0.00, 0.02]	0.24 [0.21, 0.27]	0.28 [0.22, 0.34]	0.18
Caffeine	0.18 [0.16, 0.19]	0.08 [0.08, 0.08]	0.08 [0.08, 0.08]	0.8
Alcohol	0.28 [0.27, 0.28]	0.18 [0.16, 0.19]	0.17 [0.14, 0.20]	0.67

Data are presented as the mean and 95% confidence interval.

DII, dietary inflammatory index; MUFA, monounsaturated fatty acids; PUFA, polyunsaturated fatty acids.

^*^P value < 0.05.

**Table 3:** Logistic regression on the association between DII and infertility.

	**Non-adjusted model**		**Model I**		**Model II**	
	**OR [95% CI]**	***P*** **-value**	**OR [95% CI]**	***P*** **-value**	**OR [95% CI]**	***P*** **-value**
Continuous DII	1.09 [1.01, 1.17]	0.04^*^	1.09 [1.01, 1.18]	0.03^*^	1.09 [1.01, 1.18]	0.04^*^
DII-Q1	Reference	-	Reference	-	Reference	-
DII-Q2	1.37 [0.94, 1.98]	0.1	1.42 [0.98, 2.08]	0.07	1.51 [1.00, 2.28]	0.05^*^
DII-Q3	1.09 [0.78, 1.53]	0.6	1.09 [0.76, 1.57]	0.62	1.07 [0.73, 1.57]	0.74
DII-Q4	1.61 [1.12, 2.31]	0.01^*^	1.66 [1.14, 2.40]	0.01^*^	1.71 [1.17, 2.51]	< 0.001^***^

Data are presented as OR [95% confidence interval]. Model I adjusted for age, marital status and race/ethnicity. Model II adjusted for age, marital status and race/ethnicity, education levels, family income, BMI, regular menstrual periods, pelvic infection, female hormones taken, birth control pills taken, drinking history and smoking history.

BMI, body mass index; DII, dietary inflammation index.

^***^P-value < 0.001.

^*^P-value < 0.05.

**Figure 2:**
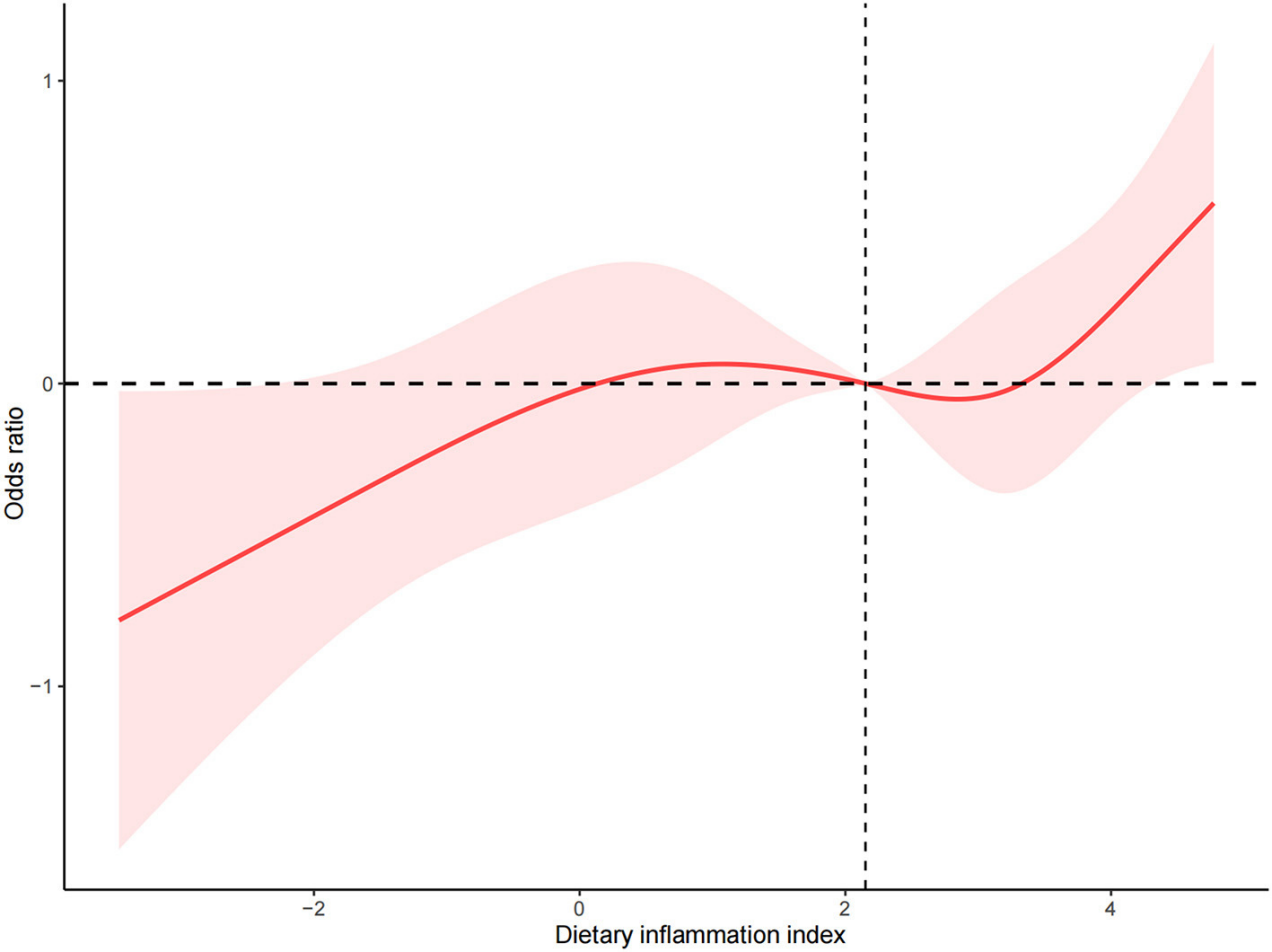
Association of Dietary Inflammatory Index and infertility.

The relationship between predicted infertility risk and DII was effectively modeled and visualized using RCS, as illustrated in Figure 2. In this study, we found that when DII is < 2.4, the increase in infertility rates is relatively slow with the elevation of DII. However, when it exceeds 2.4, the infertility rate increases more significantly with the rise of DII. Therefore, proper control of DII is crucial for preventing infertility.

### Subgroup analysis

**Figure 3:**
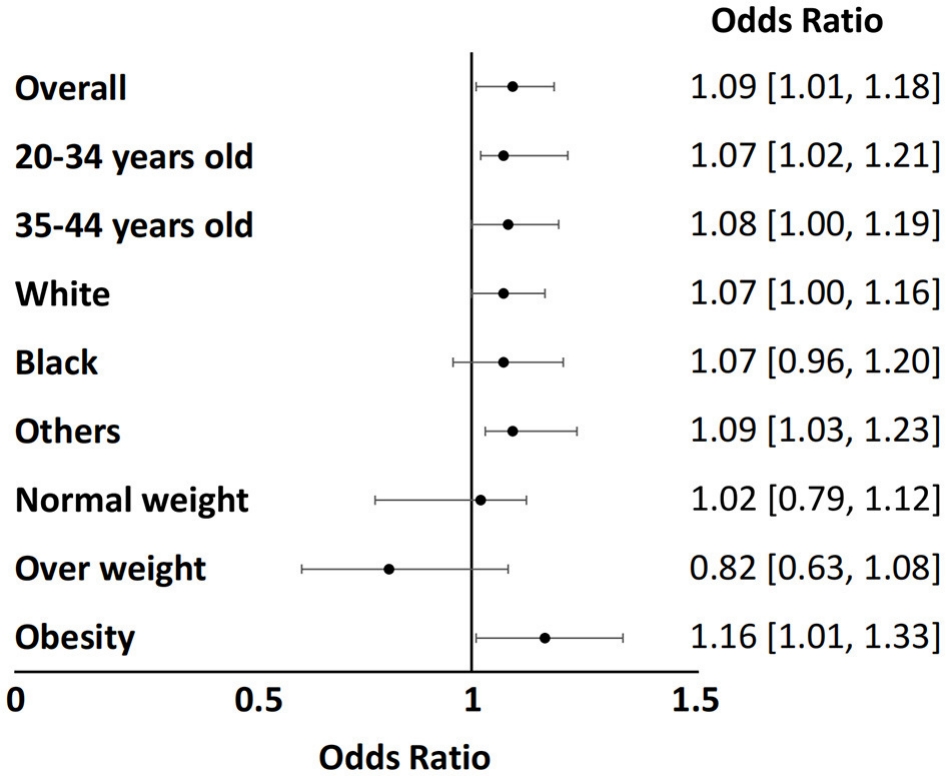
Subgroup analysis of the correlation between Dietary Inflammatory Index and infertility.

Subgroup analysis was conducted using stratified logistic regression, categorizing participants by age, race/ethnicity, and BMI. The forest plot (Figure 3) revealed that obese patients had a higher risk of infertility (OR = 1.16, 95% CI = 1.01–1.33). Despite this finding, likelihood ratio tests showed that there were no significant interactions between age, race/ethnicity, and the association between DII and infertility.

## Discussion

This cross-sectional study aimed to explore the relationship between Dietary Inflammatory Index (DII) and infertility. After accounting for potential confounding factors, the results indicated that individuals consuming a diet with higher inflammatory properties, as measured by DII, had a significantly higher prevalence of infertility. The study demonstrated that with each increase in DII score, the likelihood of experiencing infertility increased.

In recent years, there has been a rising trend in the incidence of infertility, which has been significantly impacting individuals of childbearing age and imposing a burden on society (13). Inflammation has emerged as a key factor contributing to poor reproductive outcomes, commonly referred to as “inflammatory infertility” (7). Numerous studies have established a link between inflammatory conditions and an elevated risk of infertility, such as pelvic inflammatory disease, endometriosis, and polycystic ovary syndrome (14). Systemic inflammation can adversely affect the uterus, cervix, and placenta, thereby decreasing fertility (15).

Decades ago, researchers observed that women following a “fertility diet” may have a higher likelihood of becoming pregnant and ovulating (16). Recent studies have indicated that infertile women undergoing *in vitro* fertilization (IVF) and adhering to a Mediterranean diet may experience improved pregnancy outcomes (17, 18). Western dietary patterns, known for their high fat and calorie content, have been associated with elevated levels of CRP and IL-6, leading to heightened systemic inflammation (19). A cross-sectional study demonstrated that consuming a pro-inflammatory diet is associated with an 86% higher likelihood of infertility in women, a relationship that remained statistically significant even after adjusting for confounding variables with odds ratio of 76% (20). While the exact ways in which anti-inflammatory components impact fertility outcomes are not completely understood, it is suggested that diet, as a modifiable lifestyle factor, could play a crucial role in the treatment of inflammation-related diseases.

DII plays a significant role in influencing the female reproductive system by impacting the body's inflammatory status. Studies have shown that a diet high in DII can result in elevated levels of inflammatory markers like CRP and interleukins. These increased levels have the potential to disturb the secretion of ovarian hormones, hinder the maturation and release of eggs, and affect endometrial angiogenesis and cell proliferation. As a result, this reduced endometrial receptivity may hinder the successful implantation of embryos (9). Moreover, a high DII diet can affect female fertility through metabolic pathways, such as insulin resistance and obesity triggered by excessive consumption of red and processed meats. These factors can compromise ovarian function and disrupt the hormonal balance, thereby escalating the risk of infertility (25). On the other hand, a low DII diet is characterized by the consumption of anti-inflammatory foods and nutrients that help reduce inflammation in the body. These antioxidant-rich foods help support reproductive system health by reducing oxidative stress and combating free radicals. Reducing inflammation with a low-DII diet could improve ovarian function, boost endometrial receptivity, and decrease the risk of fallopian tube blockage (21).

Despite the existing literature, there is limited research exploring the relationship between DII and infertility. A recent cross-sectional study encompassing 4,437 participants indicated that adherence to a pro-inflammatory diet, as indicated by a higher DII, was associated with a 76% increased risk of infertility in women (20). Conversely, a prospective observational study conducted by Diba-Baghtash et al. reported no significant association between DII and pregnancy outcomes in infertile women undergoing IVF (22). However, given the relatively small sample size of this study (*n* = 144), the findings should be interpreted with caution. Therefore, further research, including clinical trials with human participants, is essential to achieve a more comprehensive understanding of this relationship.

In addition to the physiological effects, the relationship between DII and female infertility could also involve psychological and social aspects (23). Infertility often causes significant psychological stress in women, leading to problems such as anxiety and depression. Poor mental health can further impact the endocrine system and worsen infertility symptoms. In such situations, dietary modifications to reduce DII levels may help alleviate psychological distress in women, indirectly benefiting fertility.

Reducing the intake of pro-inflammatory foods and increasing the consumption of anti-inflammatory foods can enhance the body's inflammatory environment, thus benefiting the reproductive system's health. This dietary shift not only aids in preventing infertility but also effectively boosts women's fertility. Therefore, incorporating dietary interventions as a critical element in strategies for preventing and treating infertility is of paramount importance for safeguarding women's reproductive health. By making sensible dietary modifications, we can offer substantial backing for women's reproductive health and achieve a more holistic approach to health management.

This study also has some limitations. Firstly, the infertility assessments were based on self-reported data, which while a valid method, may be influenced by factors such as the male partner's infertility and memory recall issues regarding the timing of conception, potentially leading to biased results. Additionally, some participants may have altered their dietary habits after being diagnosed with infertility. Thirdly, due to the cross-sectional nature of the NHANES study, establishing a causal relationship between DII and infertility risk is challenging. In conclusion, further validation of the findings is necessary through additional prospective studies.

## Conclusion

This study, which encompassed a nationally representative sample, identified a positive association between increased consumption of a pro-inflammatory diet, as measured by a higher DII score, and the risk of infertility in American adults. The findings of the study imply that lowering DII levels through dietary interventions could potentially mitigate inflammation, enhance reproductive health, improve female fertility, and yield positive outcomes on various socio-demographic variables. However, infertility is a complex issue influenced by multiple factors, requiring a holistic approach and individualized treatment strategies. Additional research is warranted to establish a robust evidence base regarding the specific correlation between DII and female infertility.

## Data Availability

The original contributions presented in the study are included in the article, further inquiries can be directed to the corresponding authors.
