# Supplementary material for: Dietary Inflammatory Index and female infertility: findings from NHANES survey
Source: Front Nutr. 2024 Sep 19;11:1391983. doi: 10.3389/fnut.2024.1391983 (PMC11446885; doi:10.3389/fnut.2024.1391983)
Supplement: Supplementary file 1 [file Table_1.docx]

**Table S1. Baseline Characteristics.**

|  | **Overall (n = 3071)** | **DII-Q1** | **DII-Q2** | **DII-Q3** | **DII-Q4** | ***p* value** |
| --- | --- | --- | --- | --- | --- | --- |
| **Age, years** | 31.18 [30.80, 31.57] | 31.65 [31.04, 32.26] | 30.80 [29.92, 31.68] | 31.33 [30.69, 31.98] | 30.91 [30.45, 31.38] | 0.21 |
| **Race/ethnicity** |  |  |  |  |  | <0.001*** |
| **White** | 56.38 [48.75, 64.00] | 57.93 [52.95, 62.91] | 55.87 [50.00, 61.75] | 57.27 [50.16, 64.37] | 54.16 [48.41, 59.92] |  |
| **Black** | 13.26 [10.81, 15.71] | 9.27 [ 6.58, 11.95] | 11.40 [ 8.51, 14.30] | 14.10 [10.98, 17.23] | 18.86 [14.53, 23.20] |  |
| **Mexican** | 11.79 [ 8.88, 14.71] | 13.06 [ 9.56, 16.57] | 14.93 [10.74, 19.12] | 9.86 [ 6.79, 12.93] | 9.09 [ 6.27, 11.90] |  |
| **Other Hispanic** | 8.05 [6.33, 9.77] | 8.44 [6.67, 10.20] | 7.39 [5.30, 9.49] | 8.01 [5.00, 11.02] | 8.39 [5.81, 10.96] |  |
| **Others** | 10.52 [8.98, 12.06] | 11.30 [8.51, 14.09] | 10.40 [8.49, 12.32] | 10.76 [7.38, 14.13] | 9.50 [7.39, 11.61] |  |
| **Education levels** |  |  |  |  |  | <0.001*** |
| **Less than high school** | 3.26 [2.37, 4.16] | 3.04 [1.74, 4.35] | 3.22 [1.99, 4.45] | 4.14 [2.62, 5.67] | 2.56 [1.38, 3.73] |  |
| **High school or equivalent** | 28.03 [24.70, 31.36] | 18.35 [14.81, 21.89] | 27.62 [23.86, 31.38] | 27.14 [22.33, 31.95] | 40.47 [34.76, 46.18] |  |
| **College or above** | 68.68 [61.84, 75.52] | 78.61 [74.37, 82.84] | 69.16 [64.82, 73.50] | 68.72 [63.40, 74.03] | 56.97 [51.27, 62.68] |  |
| **Marital status, *n* (%)** |  |  |  |  |  | 0.02* |
| **Divorced** | 6.09 [ 4.91, 7.27] | 5.85 [3.49, 8.21] | 5.48 [3.54, 7.43] | 5.68 [3.37, 7.99] | 7.49 [5.47, 9.51] |  |
| **Living with partner** | 14.69 [12.68, 16.69] | 12.14 [ 8.67, 15.61] | 14.72 [11.90, 17.53] | 15.13 [11.55, 18.72] | 17.02 [13.91, 20.12] |  |
| **Married** | 44.16 [39.56, 48.76] | 50.96 [46.35, 55.57] | 44.15 [38.84, 49.46] | 43.65 [38.89, 48.41] | 37.08 [32.31, 41.85] |  |
| **Never married** | 31.62 [28.52, 34.72] | 28.69 [24.52, 32.87] | 33.18 [28.57, 37.79] | 31.32 [27.45, 35.19] | 33.54 [29.04, 38.04] |  |
| **Separated** | 3.18 [ 2.48, 3.87] | 2.11 [1.05, 3.18] | 2.12 [1.25, 2.99] | 3.93 [2.44, 5.42] | 4.69 [3.03, 6.35] |  |
| **Widowed** | 0.27 [ 0.07, 0.47] | 0.24 [0.04, 0.52] | 0.36 [ 0.04, 0.67] | 0.28 [0.11, 0.68] | 0.19 [0.08, 0.46] |  |
| **Family income** |  |  |  |  |  | 0.002** |
| **< 2000$** | 17.44 [15.42, 19.46] | 13.66 [10.84, 16.49] | 16.79 [13.53, 20.04] | 20.14 [16.91, 23.38] | 22.32 [18.21, 26.42] |  |
| **≥ 2000$** | 78.76 [72.28, 85.23] | 86.34 [83.51, 89.16] | 83.21 [79.96, 86.47] | 79.86 [76.62, 83.09] | 77.68 [73.58, 81.79] |  |
| **BMI, kg/m^2^** | 29.38 [28.92, 29.84] | 28.02 [27.30, 28.74] | 29.21 [28.28, 30.13] | 29.70 [29.03, 30.38] | 30.73 [29.92, 31.54] | <0.001*** |
| **Regular menstrual periods, (%)** | 90.12 [83.59, 96.64] | 93.21 [90.47, 95.94] | 90.58 [87.23, 93.92] | 90.89 [88.16, 93.62] | 85.24 [82.86, 87.62] | 0.01* |
| **Pelvic infection, (%)** | 4.47 [ 3.46, 5.48] | 2.74 [1.28, 4.21] | 4.65 [3.10, 6.21] | 4.67 [2.79, 6.54] | 6.10 [4.24, 7.97] | 0.06 |
| **Female hormones taken, %** | 4.46 [ 3.11, 5.81] | 3.96 [1.91, 6.01] | 4.65 [2.29, 7.02] | 5.08 [2.48, 7.68] | 4.16 [2.08, 6.23] | 0.86 |
| **Birth control pills taken, %** | 72.92 [66.43, 79.41] | 75.76 [72.21, 79.31] | 72.90 [69.15, 76.65] | 73.84 [70.47, 77.21] | 69.01 [64.80, 73.21] | 0.05* |
| **Smoking, %** | 19.75 [17.29, 22.22] | 12.00 [ 9.27, 14.72] | 14.36 [11.16, 17.56] | 23.10 [18.63, 27.57] | 30.69 [26.61, 34.77] | <0.001*** |
| **Drinking, %** | 83.64 [77.24, 90.04] | 87.35 [83.52, 91.18] | 86.77 [83.49, 90.05] | 86.38 [82.34, 90.41] | 85.44 [82.41, 88.47] | 0.84 |

Continuous variables are presented as the mean and 95% confidence interval, category variables are presented as the proportion and 95% confidence interval. BMI, body mass index. *** *P* value<0.001, ** *P* value<0.01, * *P* value<0.05.
